# Deletion of a single glycosyltransferase in *Caldicellulosiruptor bescii* eliminates protein glycosylation and growth on crystalline cellulose

**DOI:** 10.1186/s13068-018-1266-x

**Published:** 2018-09-24

**Authors:** Jordan Russell, Sun-Ki Kim, Justin Duma, Harald Nothaft, Michael E. Himmel, Yannick J. Bomble, Christine M. Szymanski, Janet Westpheling

**Affiliations:** 1 Microbiology Department, University of Georgia, Athens, GA USA; 2 Genetics Department, University of Georgia, Athens, GA USA; 3 Department of Food Science and Technology, Chung-Ang University, Anseong, Gyeonggi 17546 Republic of Korea; 4 Complex Carbohydrate Research Center, University of Georgia, Athens, GA USA; 5 Department of Biological Sciences, University of Alberta, Edmonton, AB Canada; 6 Biosciences Center, National Renewable Energy Laboratory, Golden, CO USA; 7 The BioEnergy Science Center and The Center for Bioenergy Innovation U.S. Department of Energy Office of Science, Oak Ridge, Tennessee USA

## Abstract

Protein glycosylation pathways have been identified in a variety of bacteria and are best understood in pathogens and commensals in which the glycosylation targets are cell surface proteins, such as S layers, pili, and flagella. In contrast, very little is known about the glycosylation of bacterial enzymes, especially those secreted by cellulolytic bacteria. *Caldicellulosiruptor bescii* secretes several unique synergistic multifunctional biomass-degrading enzymes, notably cellulase A which is largely responsible for this organism’s ability to grow on lignocellulosic biomass without the conventional pretreatment. It was recently discovered that extracellular CelA is heavily glycosylated. In this work, we identified an O-glycosyltransferase in the *C. bescii* chromosome and targeted it for deletion. The resulting mutant was unable to grow on crystalline cellulose and showed no detectable protein glycosylation. Multifunctional biomass-degrading enzymes in this strain were rapidly degraded. With the genetic tools available in *C. bescii*, this system represents a unique opportunity to study the role of bacterial enzyme glycosylation as well an investigation of the pathway for protein glycosylation in a non-pathogen.

**Electronic supplementary material:**

The online version of this article (10.1186/s13068-018-1266-x) contains supplementary material, which is available to authorized users.

## Introduction

Bacteria of the thermophilic genus *Caldicellulosiruptor* are of industrial interest for their ability to efficiently degrade crystalline cellulose and to utilize lignocellulosic biomass without the need for the conventional pretreatment [1, 2]. As the tools for genetic manipulation have been developed in one of the most cellulolytic species, *C. bescii* [3, 4], this organism has been explored both as a potential candidate for consolidated bioprocessing (CBP) [5, 6] and as a source for novel, thermophilic lignocellulose-degrading enzymes [7, 8]. *C. bescii* secretes a suite of biomass-degrading enzymes, the most prevalent being multifunctional in nature, the most abundant of which is Cellulase A [9] that plays an essential role in the cellulolytic activity of the exoproteome [10]. Most of these multifunctional enzymes consist of two glycoside hydrolases and several carbohydrate-binding modules (CBMs) connected by linker peptide regions [11], CelA is one of several such tethered, multifunctional enzymes expressed by *C. bescii* [8] and is so far the single most cellulolytic gene product ever isolated from a microorganism [12, 13]. In addition, we recently showed that the extracellular form of CelA is heavily glycosylated [14].

Though it was long thought that bacteria did not make glycoproteins, protein glycosylation pathways are now being discovered in a wide variety of bacteria including pathogens and commensals, and these bacteria utilize a wider array of pathways and sugars than eukaryotes. There are two main types of protein glycosylation: N-linked glycosylation of the amide nitrogen on Asn residues, and O-linked glycosylation of the hydroxyl oxygen on typically on Ser and Thr residues [15]. Archaea, bacteria, and eukaryotes possess N-linked and O-linked protein glycosylation machineries; however, some bacteria also have specialized glycosylation systems (such as the adhesion-specific glycosylation systems in *E. coli* and *Haemophilus influenzae*) [15, 16]. Bacterial glycosylation has been studied primarily in the context of cell surface proteins, such as the S layers, pili, and flagella of pathogens. The bacterial N-glycan pathway is best characterized in *C. jejuni* where a conserved glycan is added to specific asparagine residues in the periplasm by the oligosaccharyltransferase (OTase), the N-OTase PglB. In *C. jejuni,* loss of N-glycosylation reduces the colonization potential in chickens and mice [17, 18], diminishes the ability to adhere to and invade intestinal epithelial cells in vitro [18], results in decreased DNA uptake [19], and increases susceptibility to gut proteases [20]. In contrast, *Neisseria* species possess an O-linked glycosylation system that results in the transfer of a glycan to serine (S) residues of select periplasmic proteins by the O-OTase PglL [21]. Another O-linked system in the rising nosocomial pathogen *Acinetobacter baumannii*, driven by the O-OTase PglC, is responsible for capsule biosynthesis, and its disruption weakens biofilm formation and attenuates virulence in mice [22]. Although unusual for *O*-glycosylation, in these cases, both the bacterial O-linked and N-linked systems build the oligosaccharide as a lipid-linked precursor (a polyprenyl-linked intermediate or LLO) on the cytoplasmic side of the inner membrane that is then flipped into the periplasmic space and transferred *en bloc* to target proteins by the respective OTases [21, 23]. Whereas, in bacteria, the pathways are not essential for viability, glycosylation deficiencies and defects in protein N-glycosylation in eukaryotes result in more severe phenotypes, classified as congenital disorders of glycosylation in mammals [24].

In contrast, relatively little is understood about the impact of glycosylation on secreted bacterial enzymes. While previous work analyzed the impact of glycosylation specifically on fungal cellulase enzymes [25], the results varied greatly in terms of enzyme activity, binding affinity, thermostability, susceptibility to cleavage, and protein transport. For the well-characterized *Trichoderma reesei* cellulase, Cel7A, O-glycosylation of the linker peptides enhances substrate affinity [26] and enhances resistance to proteolysis [27]. This proteolysis protection has also been demonstrated for bacterial cellulases in *Cellulomonas fimi* [28]. Sequence analysis of CelA, using the GlycoPP webserver [29], indicated favorable sites for N-linked glycosylation in both the GH9 and GH48 domains and sites for O-linked glycosylation spanning the linker regions and CBMs [14]. The linkers of CelA consist primarily of alternating Thr and Pro residues with Ser residues always found near the interface with CBMs and catalytic regions; the same also holds for the other multifunctional enzymes in the exoproteome. In this work, we took an in vivo approach to begin to dissect the pathway for, and impact of, glycosylation in *C. bescii*. A glycosyltransferase family 39 gene, likely to be involved in protein glycosylation, was identified bioinformatically and deleted by marker replacement. A periodic acid Schiff (PAS) glycoprotein stain revealed that the extracellular enzymes secreted by the resulting GT39 mutant were devoid of any glycosylation. Western analysis of CelA revealed that it is cleaved to a far greater degree in the absence of glycosylation when compared to wild type. Growth curves on cellobiose and Avicel reveal that glycosylation is essential to the ability of *C. bescii* to digest crystalline cellulose, but not to growth on simple sugars. An understanding of the glycosylation pathway in *C. bescii* will provide new insight into related systems in other bacteria and could guide future heterologous expression system design for CelA and other thermophilic enzymes.

## Results and discussion

### Bioinformatic analysis identified a putative glycosyltransferase located in close proximity to gene-encoding prominent cellulolytic enzymes, including CelA

**Fig. 1:**
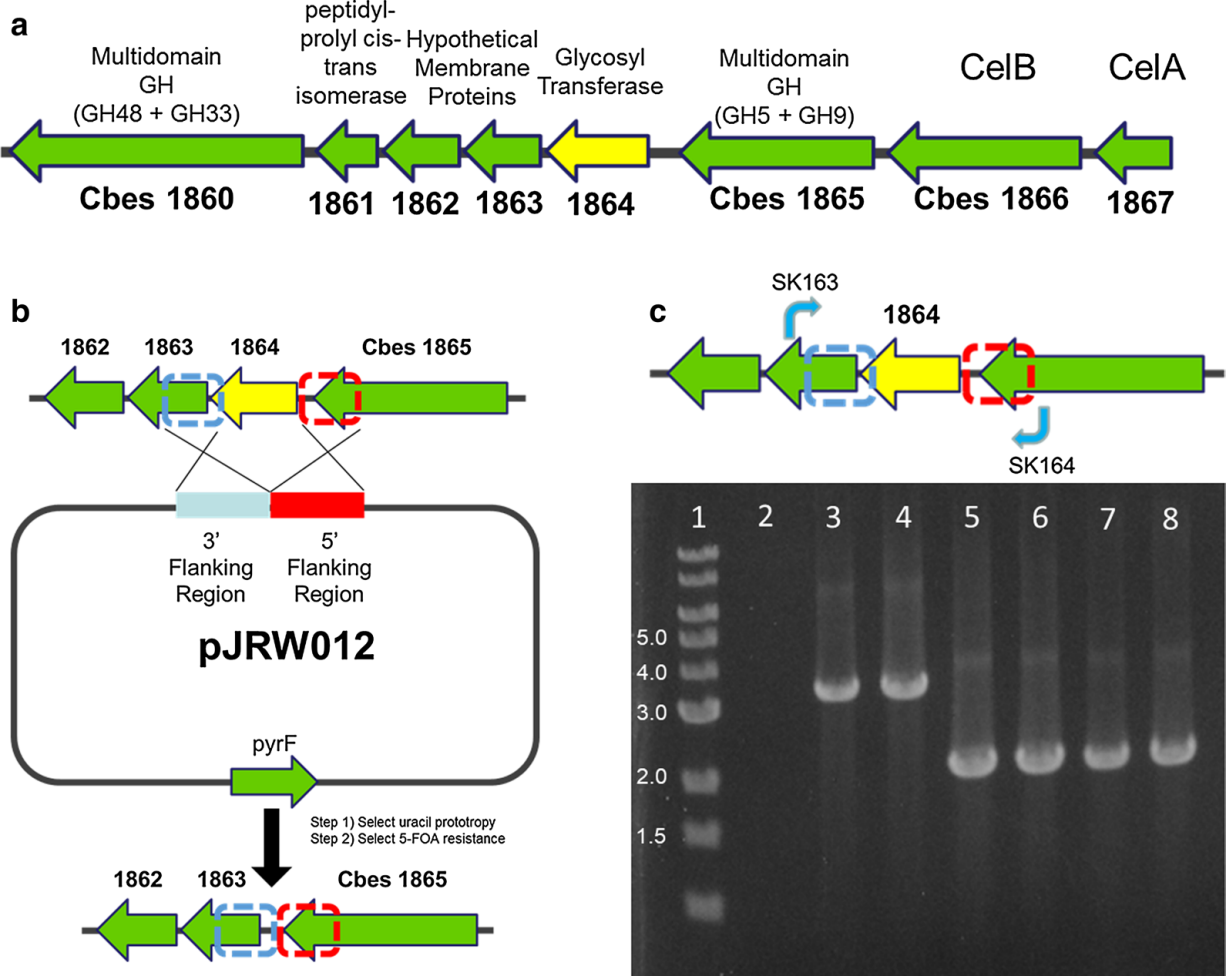
Deletion of glycosyltransferase in *C. bescii.* **a** Chromosome map of the glycosyltransferase (Cbes1864) and surrounding genes. **b** Depiction of the deletion cassette consisting of a fused 5′ and 3′ flanking region in a non-replicating plasmid, pJRW012, with a copy of the *pyrF* gene from *Clostridium thermocellum* (Clo1313_1266) for selection of uracil prototrophic transformants of a *ΔpyrF* background strain. Counterselection with 5-FOA selected strains that had undergone a second recombination event resulting in strain JWCB143 (*ΔpyrF ΔcbeI Δcbes1864*). **c** Agarose gel showing PCR products amplified with primers SK163 and SK164 on gDNA templates from wild-type *C. bescii* (lane 3), parent strain JWCB018 (lane 4), Δcbes1864 strain JWCB143 (lane 5), and no template (lane 2). Lane 1: DNA molecular weight standards, Lanes 6–8: JWCB143 sister isolates. Expected band for wild-type locus is 3.7 kb and for cbes1864 deletion locus is 2.2 kb

Biosynthesis of saccharides involves the action of different glycosyltransferases (GTs) with a remarkable diversity in their donor, acceptor, and product specificity. To date, more than 35,000 GTs have been classified into families based on sequence, fold, and mechanistic similarities [30]. However, for some enzymes, classification is difficult and, for over 7000 GTs, a classification is not yet available. Prominent protein-modifying GTases belong to the following CAZY families [30]. Family GT66-inverting enzymes use lipid-diphospho-oligosaccharide donors for N-glycosylation of proteins and include members such as PglB in *C. jejuni*, STT3 in eukaryotes, and AglB in Archaea. Members of the inverting family GT39 are responsible for O-linked glycosylation through the use of lipid-phospho-carbohydrate donors as described in *Mycobacteria tuberculosis* [31] and *Streptomyces coelicolor* [32]. In contrast, the HMWC1 glycosyltransferase in *H. influenzae* groups with family GT41, but forms a novel class of N-glycosyltransferases [33]. Some GTases cannot be grouped such as PglL, responsible for the unique en bloc *O*-linked protein glycosylation in *Neisseria* species. A primary screen of the *C. bescii* GT content using the CAZY database [34] revealed 56 hits for carbohydrate-modifying enzymes. No GT66 or GT41 members were identified, but one family GT39 was identified encoded by Cbes_1864. Blast searches (BlastP, NCBI) confirmed the GT39 family designation of Cbes_1864 and the homology to membrane-bound dolichyl-phosphate-mannose-protein mannosyltransferases, further indicating that Cbes_1864 might be using a lipid-linked sugar rather than a nucleotide-activated sugar donor. Proteins from this family are responsible for O-linked glycosylation of proteins and catalyze the reaction: dolichyl-phosphate d-mannose + protein → dolichyl phosphate + O-D-mannosyl-protein, a process that is well characterized in *Saccharomyces cerevisiae* and in higher eukaryotes [35]. Interestingly, Cbes_1864 is located close to *celA* encoding the potential acceptor, within a region of the *C. bescii* genome previously described as the Glucan Degradation Locus [36] (Fig. 1a). The genes Cbes_1867 to Cbes_1860 are in the same orientation, and the GT39 gene, Cbes_1864, is part of a four-gene cluster (Cbes_1861 to Cbes_1864) with short intergenic regions, potentially indicating a co-transcribed operon structure. Cbes_1864 consists of 556 amino acids, with a predicted MW of 65 kDa and a pI of 9.74, indicating that Cbes_1864 is an integral membrane protein [37]. Further domain structure analysis by PHYRE revealed that the C-terminal part (59% of the sequence, aa 169–558 of Cbes_1864) has structural homology to a transferase from *Cupriavidus metallidurans* bound to undecaprenyl phosphate (100% confidence), to the membrane domain of the oligosaccharyltransferase from *Archaeoglobus fulgidus* (99.8% confidence) and to the membrane domain of the oligosaccharyltransferase, and PglB from *Campylobacter lari* (99.8% confidence). Topology analysis further predicts that Cbes_1864 possesses 10–12 (depending on the program used) transmembrane helices with the N- and the C-termini predicted to face the extracellular space. The N-terminus is further predicted to contain a signal peptide (program Phobius [38]*)* to target the protein to the membrane and the N-terminal domain (aa 32–124 located in loop 1) structurally aligns with sugar-binding proteins/hydrolases and proteins that possess a “general” carbohydrate-binding module (with 97.4 to 96.0% confidence). In summary: in silico analysis identified Cbes_1864, a family GT39 glycosyltransferase with structural homology to the archaeal and bacterial O-Tases most likely using a lipid-linked (dolichyl- or undecaprenyl phosphate-linked) sugar donor substrate, as a potential GT protein responsible for (O-linked) glycosylation of CelA. Examination of the websites listing organisms capable of plant polysaccharide degradation revealed that Cbes_1864 exists in the genomes of most cellulose-degrading microbes, including those capable of cellulosome formation, such as *Clostridium thermocellum*.

### Deletion of a glycosyltransferase family 39 gene results in a loss of protein glycosylation

To generate a deletion of Cbes_1864 in the *C. bescii* chromosome, vector pJRW012 (Additional file 1: Fig. S1) was constructed for the targeted deletion of 1500 bases in the 5′-prime end of the 1701 base pair open-reading frame by joining 1 kb regions upstream and downstream of this region for homologous recombination and marker replacement. The last 200 bp of the 3′-prime ORF sequence was retained to avoid the disruption of potential regulatory sequences for the adjacent gene, Cbes1863. Plasmid pJRW012 contains a wild-type allele of the *pyrF* gene from *Clostridium thermocellum* (Clo1313_1266) and does not contain an origin of replication for *C. bescii*. pJRW012 was transformed into *C. bescii* JWCB018 that contains a deletion of the *pyrFA* gene, rendering it a uracil auxotroph. Transformants were selected for uracil prototrophy and plasmid integration at the Cbes1864 locus is shown in (Fig. 1b). Counter selection of the *pyrF* wild-type allele with 5-fluoroorotic acid (5-FOA), which is converted to the toxic 5-fluorouracil in the presence of the wild-type *pyrF* allele, was used to select for the elimination of plasmid DNA. PCR with primers binding upstream and downstream of the open-reading frame as well as outside of the flanking regions for integration were used to screen for deletion of Cbes1864 (Fig. 1c). Deletion resulted in a 2.21-kb fragment, distinguishable from the 3.70-kb wild-type fragment followed by sequencing of the 2.21-kb PCR product. Additional PCR screening with one or both primers inside the Cbes1864 reading frame was also performed, producing the expected fragment for the wild-type strains and not from the deletion strain (Additional file 1: Figs. S2, S3).

**Fig. 2:**
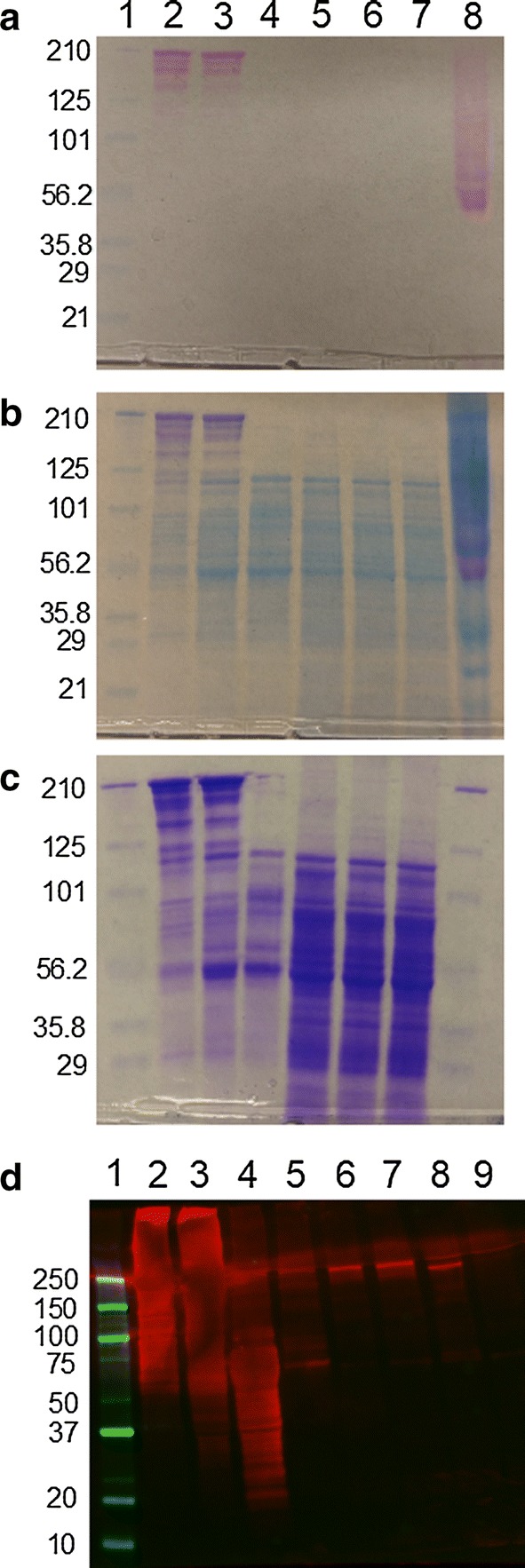
Effect of the glycosyltransferase deletion on protein glycosylation and CelA stability. **a**, **b,** and **c** are SDS-PAGE gels and the protein loadings are the same. Molecular weight standards (lane 1), JWCB001 (wild type) ECP (lane 2), JWCB018 (*ΔpyrF* parent strain) ECP (lane 3), JWCB143 (glycosyltransferase deletion) ECP (lane 4), JWCB001 (wild type) ICP (lane 5), JWCB018 (*ΔpyrF* parent strain) ICP (lane 6), and JWCB143 (glycosyltransferase deletion) ICP (lane 7). **a** Stained with glycoprotein stain. Lane 8 is a glycosylation positive standard. **b** The same gel counterstained with RAPIDstain to visualize protein. **c** The same protein fractions run on an SDS-PAGE gel stained with Coomassie Brilliant Blue without prior glycoprotein stain. **d** Western Blot. molecular weight standards (lane 1), JWCB001 ECP (lane 2), JWCB018 ECP (lane 3), JWCB143 ECP (lane 4), JWCB029 ECP (lane 5), JWCB001 ICP (lane 6), JWCB018 ICP (lane 7), JWCB143 ICP (lane 8), and JWCB029 ICP (lane 9). CBM3c was the primary antibody with an HRP-linked secondary antibody, visualized by chemiluminescence

To examine the effects of the deletion on *C. bescii* protein glycosylation, extracellular protein was concentrated from cell supernatants and intracellular proteins were obtained as lysates from the corresponding pellets from the wild-type (JWCB001), parent (JWCB018), and mutant (JWCB143) strains. Extracellular and intracellular fractions were separated on an SDS-PAGE gel and stained using a Glycoprotein Staining Kit (Fig. 2a). As observed previously [14], only proteins in the extracellular fraction reacted with this stain in JWCB001 and JWCB018, including the characteristic CelA band and several other distinct high-molecular-weight (> 125 kDa) bands corresponding to other multifunctional enzymes. No glycoproteins were detected in the JWCB143 extracellular fraction. Counter staining of the gel visualized the protein content in each lane (Fig. 2b). The high-molecular-weight extracellular protein bands that stained positive for glycans in the JWCB001 and JWCB018 lane were absent from JWCB143. Additional SDS-PAGE analysis with the same protein fractions confirmed that the absence of high-molecular-weight extracellular proteins was not an artifact of the glycostaining reaction (Fig. 2c). These results indicate that Cbes_1864 is essential for glycosylation in *C. bescii*, and are consistent with the previous observations in which only extracellular version of CelA was glycosylated [14].

### CelA is secreted in the absence of glycosylation, but is unstable in the cell supernatant

A western blot of the intracellular and extracellular fractions of JWCB001, JWCB018, JWCB143, and the Δ*celA* strain JWCB029, using monoclonal anti-CBM3c antibodies, tracked CelA from these strains (Fig. 2d). An array of bands was present in all three extracellular fractions containing CelA, as expected, since CelA is known to exist both in an intact, full-length form as well as in several truncated forms in wild-type cell supernatants [12]. In the glycosyltransferase deletion strain (JWCB143), the array of CelA bands is markedly shifted to molecular weights below 100 kDa when compared to the wild-type and parent strains, indicating that, in the absence of glycosylation, CelA is susceptible to increased degradation or cleavage. This is consistent with past work associating protein glycosylation with proteolytic protection of cellulases in both fungi and bacteria [26, 27]. This result explains the disappearance of the high-molecular-weight bands from the JWCB143 extracellular fraction in (Fig. 2c), a distinct phenotype that may allow a simple screen for other glycosylation-related *C. bescii* mutants. This result also suggests that glycosylation is not required for protein transport as CelA is present in the extracellular fraction of JWCB143 at similar apparent densities as in the parent and background strains. In the intracellular fractions, the JWCB143 CelA band migrates at a lower molecular weight, as previously observed for CelA expressed without a signal peptide [14]. These observations for CelA seem to hold for other high MW enzymes (Fig. 2a–c), as all high MW bands disappear in both the coomassie and glycostained gels. This is not surprising given the similar sequence and structure of these other multidomain enzymes, especially in the linker regions.

### Deletion of glycosyltransferase family 39 gene causes no general growth defect, but does impact the ability of *C. bescii* to grown on crystalline cellulose

**Fig. 3:**
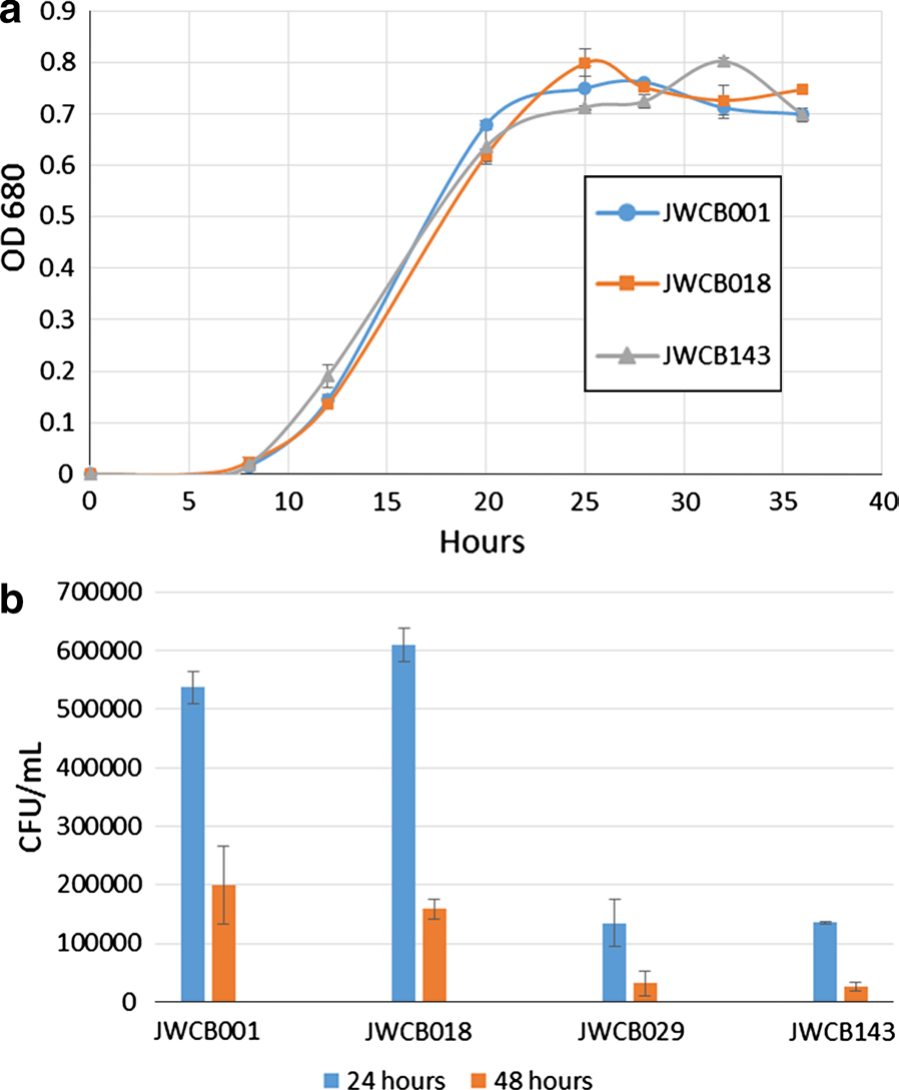
Growth of the glycosyltransferase mutant on soluble and insoluble substrates. Growth of the wild type, JWCB001 (blue), JWCB018, the *ΔpyrF* parent strain (orange), JWCB143, the glycosyltransferase deletion strain (gray) on cellobiose as measured by OD_680_ (**a**) or Avicel reported as colony-forming units/ml after plating and incubation at 24 and 48 h (**b**)

Given the impact of the glycosyltransferase deletion on CelA, and knowing the importance of CelA to the cellulolytic activity of *C. bescii*, we tested whether the removal of glycosylation affected the ability of the organism to deconstruct cellulose. This was tested by comparing the growth of the glycosylation mutant JWCB143 to wild-type, parent, and *ΔcelA* strains on the substrates cellobiose and Avicel. On the preferred disaccharide carbon source, cellobiose, no difference in growth was observed, indicating that the deletion did not cause a general growth defect **(**Fig. 3a). On the crystalline cellulose substrate, Avicel, pJRW143 exhibited a ~ 77% decrease (*p* = 0.041) in growth after 24 h compared to the parent strain JWCB018, while the *ΔcelA* strain, JWCB029, exhibited an almost identical ~ 78% decrease over the same time (Fig. 3b), which was consistent with the previous growth experiments for that strain. This growth defect in the glycosylation mutant JWCB143 indicates that glycosylation, presumably of CelA and similar multidomain glycoside hydrolases, contributes heavily to the ability of *C. bescii* to deconstruct cellulose.

### Complementation of the glycosyltransferase deletion restores glycosylation and glycoprotein stability

**Fig. 4:**
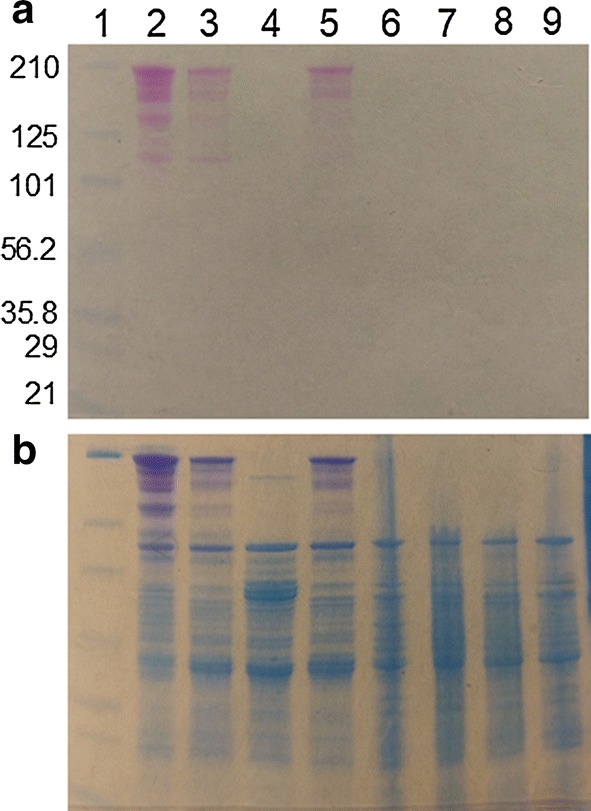
Complementation of the glycosyltransferase deletion. SDS-PAGE gel with molecular weight standards (lane 1), the wild-type strain JWCB001 ECP (lane 2), the *ΔpyrF* parent strain JWCB018 ECP (lane 3), the glycosyltransferase deletion strain JWCB143 ECP (lane 4), the complemented glycosyltransferase mutant JWCB160 ECP (lane 5) JWCB001 ICP (lane 6), JWCB018 ICP (lane 7), JWCB143 ICP (lane 8), and JWCB160 ECP (lane 9); **a** stained with Glycoprotein Staining Kit; **b** the same gel counterstained with RAPIDstain

While there is no evidence that the cluster of genes surrounding the glycosyltransferase deletion exists as an operon, the close proximity of the deletion to genes Cbes_1861–1864 (Fig. 1a) prompted us to eliminate the possibility that the deletion might have a polar effect on downstream genes. To do so, a vector, pJRW013 (Additional file 1: Fig. S4), expressing the wild type Cbes_1864 open-reading frame only was constructed using the previously described *C. bescii*/*E. coli* shuttle vector [3] for complementation of the deletion in trans from the plasmid. Notably, when transformants were plated and grown at 37 °C, the screened isolates contained only rearranged plasmids. Isolates containing the desired plasmid were only obtained when transformants were selected and incubated at room temperature (for 2 days), possibly indicating that expression of Cbes_1864 is toxic to *E. coli* cells. The presence of pJRW013 as well as the Cbes_1864 deletion in the chromosome were both confirmed by PCR (Additional file 1: Fig. S5). Protein glycosylation was restored in the complemented mutant, including glycosylation of CelA as shown by PAS staining of the JWCB160 extracellular and intracellular protein fractions (Fig. 4) and westerns using anti-CelA antibody. In addition, CelA was stable in the complemented mutant, suggesting that glycosylation is partially if not totally responsible for protein stability.

## Conclusions

The deletion of a single glycosyltransferase gene eliminated glycosylation in *C. bescii,* resulted in loss of the ability to grow on crystalline cellulose and destabilization of high-molecular-weight extracellular enzymes. The phenotype of the glycosyltransferase deletion was, in fact, the same as that of a CelA deletion mutant. Complementation with the wild-type allele restored glycosylation and enzyme stability, suggesting that a major role of this transferase is to glycosylate and stabilize long extracellular enzymes. While in vivo characterization in this work establishes the importance of glycosylation to stability and cellulolytic activity, detailed analysis of these enzymes may reveal additional contributions of glycosylation. The identification of Cbes_1864 as necessary for glycosylation in *C. bescii* is a step towards describing a novel glycosylation pathway in this hyperthermophilic Gram-positive bacterium. From an industrial perspective, this transferase may facilitate heterologous expression of these enzymes, including CelA for cost-effective production of fully functional enzymes. Past efforts to express CelA in industrial production hosts have resulted in either severe proteolytic degradation (*E. coli*) [39, 40] or substantially altered molecular weight and activity characteristics (*Bacillus megaterium*) [39]. Beyond CelA and Caldi enzymes, this transferase may also facilitate heterologous expression of other industrially relevant enzymes. Recently, a core group of CAZymes was identified (including CelA) that account for the entire cellulolytic activity of the *C. bescii* exoproteome [11]. The proposed use of this cassette to confer cellulolytic ability to other thermophiles will likely require protein glycosylation. All four of the identified enzymes are multidomain proteins with linker regions and CBMs. The presence of Cbes_1864 homologues in other cellulolytic bacteria provides an interesting foothold into studying the potential common utilization of protein glycosylation across very different native plant biomass deconstruction strategies.

## Methods

### Bacterial strains, media, and culturing conditions

Strains and plasmids used in this study are listed in Table 1. *Caldicellulosiruptor* strains were grown anaerobically on solid or liquid low osmolarity-defined (LOD) medium, pH 6.8, with maltose, cellobiose, or Avicel as the sole carbon source (all at 0.5% w/v) as indicated [41]. LOD was supplemented with uracil to a final concentration of 40 μM for the growth of uracil auxotrophic strains. Liquid cultures were grown from a 0.5% inoculum or a single colony and incubated at 65 or 75 °C in anaerobic culture bottles degassed with five cycles of vacuum and argon. *E. coli* DH5α was used for construction, preparation, and storage of plasmid DNA. *E. coli* strains were grown at 37 °C in LB broth or on LB agar supplemented with apramycin (50 µg/mL). Plasmid DNA was isolated using the QIAprep Spin Miniprep Kit (Qiagen) according to the manufacturer’s instructions. *E. coli* DH5α cells were transformed by electroporation in a 2 mm gap cuvette at 2.5 kV and selected for apramycin resistance. Chromosomal DNA from *Caldicellulosiruptor* strains was extracted using the Quick-gDNA Miniprep (Zymo) as previously described [4].

**Table 1:** Strains/plasmid used in this study

Strains/plasmids	Genotype/phenotype	Source
*C. bescii*		
JWCB001	DSMZ6725 wild type (ura^+^/5-FOA^S^)	DSMZ
JWCB018	ΔpyrFA ldh::ISCbe4 Δcbe1 (ura^−^/5-FOA^R^)	[4, 44]
JWCB029	ΔpyrFA ldh::ISCbe4 Δcbe1 ΔcelA (ura^−^/5-FOA^R^)	[10]
JWCB143	ΔpyrFA ldh::ISCbe4 Δcbe1 Δcbes1864 (ura^−^/5-FOA^R^)	This study
JWCB160	JWCB143-containing pJRW013 (ura^+^/5-FOA^S^)	This study
*E. coli*		
JW563	DH5α-containing pJRW012 (Apramycin^R^)	This study
JW626	DH5α-containing pJRW013 (Apramycin^R^)	This study
Plasmids		
pDCW173	*E. coli*/*C. bescii* shuttle vector (Apramycin^R^)	[14]
pJGW003	*C. bescii* integration vector (Apramycin^R^)	[42]
pJYW022	Expression vector for Cbes_1867 (Apramycin^R^)	This study
pJRW012	Cbes1864 deletion vector (Apramycin^R^)	This study
pJRW013	Expression vector for Cbes_1864 (Apramycin^R^)	This study

### Construction of the glycosyltransferase family 39 (Cbes1864) deletion and complementation vectors

Plasmids for this work were constructed using Q5 High-Fidelity DNA polymerase (New England BioLabs, Ipswich, MA, USA) for PCR, restriction enzymes (New England BioLabs, Ipswich, MA, USA) for digestion, and the fast-link DNA ligase kit (Epicentre Biotechnologies, Madison, WI, USA) for ligation, all according to the manufacturer’s instructions. The non-replicating integration plasmid pJRW012 for deletion of Cbes_1864 was constructed as follows. Using *C. bescii* (JWCB001) genomic DNA as template, a 5′ flanking region (1002 bp) was amplified using primers SK162 and JR022 and a 3′ flanking region (999 bp) was amplified using primers JR023 and SK161. These flanking regions were joined into one fragment (2001 bp) using overlap extension PCR (OE-PCR) with primers SK162 and SK161 adding a 5′ *Kpn*I restriction site and a 3′ *Apa*LI restriction site. A fragment-containing an apramycin resistance gene cassette, a *Clostridium thermocellum pyrF* cassette (Clo1313_1266), and the *E. coli* pSC101 replication origin was amplified from pJGW003 [42] using primers DC081 and DC262 adding the same restriction sites. The linear PCR products were digested with restriction enzymes *Kpn*I and *Apa*LI and ligated together to generate pJRW012. Ligation product was transformed into *E. coli* DH5α to generate *E. coli* JW563 and resulting plasmids were screened by diagnostic restriction digestion. The sequence of the plasmid was confirmed by automatic sequencing (Genewiz, South Plainfield, NJ, USA). The shuttle vector pJRW013 for the expression of Cbes_1864 complementation was constructed by way of an intermediate shuttle vector, pJYW022. To construct pJYW022, the entire plasmid pDCW173 [14], a CelA expression vector, was amplified by PCR using primers DC371 and JY080 adding a *Tobacco Etch Virus* protease cleavage sequence and an SphI restriction site to the 5′ end of the existing 6 residue Histidine tag. The resulting linear PCR product was digested with SphI and closed by ligation to form pJYW022 that encodes CelA with a TEV cleavable His-tag. pJRW013 was constructed by replacing the CelA-coding sequence on pJYW022 with the gene Cbes_1864 for the expression of the glycosyltransferase family 39 with a TEV cleavable His-tag. First, using *C. bescii* (JWCB001) genomic DNA as a template, the open-reading frame for Cbes_1864 was amplified with primers JR058 and JR035 (1713 bp) adding a 5′ BamHI restriction site and a 3′ SphI restriction site. Next, a backbone fragment-containing the apramycin resistance gene cassette, a *Clostridium thermocellum pyrF* cassette (Clo1313_1266), the *E. coli* pSC101 replication origin, and *C. bescii* pBAS2 replication sequence was amplified from the template pJYW022 using the primers JY081 and DC464 (7990 bp) with a 5′ SphI restriction site and a 3′ BamHI site. The two linear PCR products were digested with SphI and BamHI and ligated together to generate pJRW013. Ligation product was transformed into *E. coli* DH5α incubated at room temperature to generate *E. coli* JW626 and the resulting plasmids were screened by diagnostic restriction digestion. The sequence of pJRW013 was confirmed by Automatic sequencing (Genewiz, South Plainfield, NJ, USA).

### Deletion and complementation of the glycosyltransferase family 39 gene (Cbes1864) using a non-replicating vector

Preparations of pJRW012 isolated from *E. coli* JW563 were used to transform *C. bescii* JWCB018 by electroporation as described previously [43]. After electroporation with ~ 0.5 µg of plasmid DNA, cultures were recovered in low osmolarity complex (LOC) medium at 65 °C. Recovery cultures were transferred to LOD without uracil to select for uracil prototrophy [41]. Transformants were inoculated into non-selective LOD, with 40-µM uracil, and incubated overnight at 75 °C. Serial dilutions of this overnight culture were plated to LOD containing 4-mM 5-fluoroorotic acid (5-FOA) and 40-µM uracil as described [43]. After a 2-day incubation, colonies resistant to 5-FOA were cultured in LOD with 40 µM uracil for genomic DNA isolation and PCR screening. A PCR to screen for the deletion was performed using Jumpstart Taq DNA polymerase (Sigma-Aldrich, St. Louis, MO, USA) with primers SK163 and SK164 that were designed to hybridize outside the homologous flanking regions on the *C. bescii* chromosome. Extension time was sufficient to amplify the wild-type allele if it was still present. After the initial screening, isolates containing the expected DNA pattern were purified by two additional rounds of non-selective plating (LOD with 40-µM uracil) and PCR screening to ensure segregation of the deletion allele. The purified deletion mutant was confirmed by PCR as described above and with two additional primer pairs with one (SK163 and JR026) and then with both (JR034 and JR026) primers binding inside the targeted region of Cbes1864. The PCR product of SK163 and SK164 was sequenced to verify the site of the deletion. The verified Cbes1864 deletion strain was designated JWCB143. pJRW013 isolated from *E. coli* JW626 were used to transform *C. bescii* JWCB143 as above. Overnight cultures of *E. coli* JW626 were incubated shaking at room temperature for 2 days. Electroporation recovery cultures were transferred to LOD without uracil to select for uracil prototrophy [41]. Serial dilutions of this overnight culture were plated to LOD without uracil to maintain prototrophy. After a 2-day incubation, colonies were cultured in LOD for genomic DNA isolation and PCR screening. Genomic DNA from isolates was screened by PCR for the presence of plasmid pJRW013 using primers JR026 and DC228, and for maintenance of the Cbes_1864 deletion using primers SK163 and SK164.

### Preparation of extracellular and intracellular protein fractions

Extracellular protein (ECP) from *C. bescii* strains (JWCB001, JWCB018, JWCB029, JWCB143, and JWCB160) was collected from a 0.5–2.0-L culture grown at 65 °C in closed bottles shaking at 90 rpm to an OD_680_ of 0.25–0.3 in LOD media with cellobiose as sole carbon source and 40-mM MOPS. Cultures were centrifuged (6000×*g* at 4 °C for 15 min) and supernatants were filtered successively with 1.5- and 0.7-µm glass fiber filters to remove cells. ECP was concentrated and buffer exchanged to 20-mM MES with 2-mM β-mercaptoethanol (pH 6.0) using a 30-kDa molecular weight cut-off column (Hollow Fiber Cartridge, GE Healthcare, Chicago, IL, USA) and further concentrated using Vivaspin Turbo 15 centrifugal concentrators (Sartorius, Bohemia, NY, USA). Cell pellets were used for preparation of intracellular protein (ICP) fractions. Pellets were washed once by resuspension in 50 mL of ice-cold 50-mM Tris–Cl buffer (pH 8.0) and centrifugation (6000×*g* at 4 °C for 15 min), then resuspended in 1 mL of cell lytic B buffer (Sigma-Aldrich, St. Louis, MO, USA), and subjected to four cycles of freezing (with an ethanol and dry ice bath) and thawing (in 42 °C water bath), sonication four times for 15 s at 40 amps with 1-min rests in ice water. Lysates were centrifuged at maximum speed in microcentrifuge tubes to separate protein lysate from cell debris and the clear CFE was collected. Protein concentrations for both ECP and ICP fractions were determined using a protein assay kit (Bio-Rad, Hercules, CA, USA) with bovine serum albumin (BSA) standards.

### Detection of glycosylation and CelA protein

The ECP and ICP fractions were boiled for 15 min with SDS-cracking buffer and separated by SDS-PAGE using 4–20% gradient Mini-Protean TGX gels (Bio-Rad, Hercules, CA, USA) run at 150 V until all the loading dye was run off and then for an additional 30 min to achieve the separation of high-molecular-weight proteins. Varying protein loadings (20–60 µg) were used depending on the sample and experiment. For general protein visualization, gels were stained with Coomassie Brilliant Blue. Glycoproteins were visualized by staining with a Glycoprotein Staining Kit (G-Biosciences, St. Louis, MO, USA) according to the manufacturer’s instructions. After the initial staining and imaging of glycosylated proteins, the gel was counterstained with RAPIDstain solution from the same kit to visualize all the proteins. For detection of *C. bescii* CelA specifically, protein fractions were run on SDS-PAGE gels as described above and then transferred to a nitrocellulose membrane using a Bio-Rad Mini-Protean 3 electrophoretic unit at 100 V for 2 h. Membranes were probed with monoclonal α-CBM3C primary antibody (1:1000 dilution) and then with α-rabbit with horse radish peroxidase (HRP) secondary antibody (1:10,000). Membranes were developed with Clarity Western ECL Substrate (Bio-Rad, Hercules, CA, USA) according to the manufacturer’s instructions and imaged by chemiluminescence.

### Growth of glycosyltransferase deletion mutant on soluble and insoluble substrates

Cells were sub-cultured three times in LOD medium with 5-g/L maltose as the sole carbon source. The third sub-culture was used to inoculate 50 mL of LOD medium supplemented with 40-mM MOPS and 40-µM uracil and with 5 g/L of either cellobiose or Avicel as the sole carbon source. A 0.2% v/v inoculum was used and cultures were incubated at 65 °C with shaking at 150 rpm. Growth on cellobiose was measured by optical density (OD) at 680 nm using a Jenway Genova spectrophotometer. Growth on crystalline cellulose, Avicel PH-101, was measured by colony-forming units (CFU) by plating on LOD medium (maltose) supplemented with 40-µM uracil.

## Additional file

**Additional file 1: Figure S1.** Diagram of pJRW012, the GT39 (Cbes_1864) deletion vector. **Figure S2.** Agarose gel showing PCR products of primers JR034 and JR026, both of which bind inside of the region of Cbes_1864 targeted for deletion. **Figure S3.** Agarose gel showing PCR products of primers JR026, which binds inside of the region of Cbes_1864 targeted for deletion, and SK163 which binds to the *C. bescii* chromosome adjacent to the targeted region. **Figure S4.** Diagram of pJRW013, the GT39 (Cbes_1864) expression vector used for complementation of deleted GT39. **Figure S5.** Agarose gels showing PCR products of JR026 and DC228 which bind to expression vector pJRW013 and SK162 and SK163 which bind to the *C. bescii* chromosome flanking the Cbes_1864 region. **Table S1.** DNA primers used in this study.
