# Supplementary material for: Deletion of a single glycosyltransferase in Caldicellulosiruptor bescii eliminates protein glycosylation and growth on crystalline cellulose
Source: Biotechnol Biofuels. 2018 Sep 24;11:259. doi: 10.1186/s13068-018-1266-x (PMC6151902; doi:10.1186/s13068-018-1266-x)
Supplement: Supplementary file 1 — Additional file 1: Figure S1. Diagram of pJRW012, the GT39 (Cbes_1864) deletion vector. Figure S2. Agarose gel showing PCR products of primers JR034 and JR026, both of which bind inside of the region of Cbes_1864 targeted for deletion. Figure S3. Agarose gel showing PCR products of primers JR026, which binds inside of the region of Cbes_1864 targeted for deletion, and SK163 which binds to the C. bescii chromosome adjacent to the targeted region. Figure S4. Diagram of pJRW013, the GT39 (Cbes_1864) expression vector used for complementation of deleted GT39. Figure S5. Agarose gels showing PCR products of JR026 and DC228 which bind to expression vector pJRW013 and SK162 and SK163 which bind to the C. bescii chromosome flanking the Cbes_1864 region. Table S1. DNA primers used in this study. [file 13068_2018_1266_MOESM1_ESM.docx]

Table S1: DNA Primers used in this study

| Primer Name | Sequence 5’ 🡪 3’ |
| --- | --- |
| DC081 (w/KpnI) | AGA GAG GTA CCA CCA GCC TAA CTT CGA TCA TTG GA |
| DC222 | TAC AAG AAA AGC CCG TCA C |
| DC228 | ATC ATC CCC TTT TGC TGA TG |
| DC262 (w/ApaLI) | TGT GTG GTG CAC TCT GAC GCT CAG TGG AAC GAA |
| DC371 (w/SphI) | AGA GCA TGC TTG ATT GCC AAA CAG TAT TTC ATA TGT TGC |
| JR022 | ATC ATT ATT ACC TCG CAT TAC CTT GGC TTT ACA |
| JR023 | TGT AAA GCC AAG GTA ATG CGA GGT AAT AAT GAT TAA TTT TTC ACT CCT TTA CTT TTA CTG |
| JR026 | CAT AAT AGC TTG TCG AGA ACA GC |
| JR034 (w/SphI) | TCA TCA GCA TGC TTA GAA TAT CCA ACT CTT CAT TAT CTT TAG C |
| JR035 (w/BamHI) | TCA TCA GGA TCC ATG AAG GGA AAA AAT GTA TTA TTA TTA CTC TC |
| JR058 (w/SphI) | TCA TCA GCA TGC GAA TAT CCA ACT CCT CAT TAT CTT TAG C |
| JY080 (w/SphI) | AGA GCA TGC GAA AAC TTG TAT TTC CAG GGC CAT CAC CAT CAC CAT CAC TAA TAA TAA AGC TG |
| SK161 (w/ApaLI) | A CTA TCT GTG CAC AGC ATG TTC ATT GGC GCA AG |
| SK162 (w/KpnI) | AGA GGT ACC TGA GGT TTT GTG AAT AAT AAA TAG GCT GTT ACA |
| SK163 | ACG CCG CTT ACA ACG CAA |
| SK164 | AGT GAA GTA GCA AAT ATT ATA TCA TTG TCA AGA AGT |


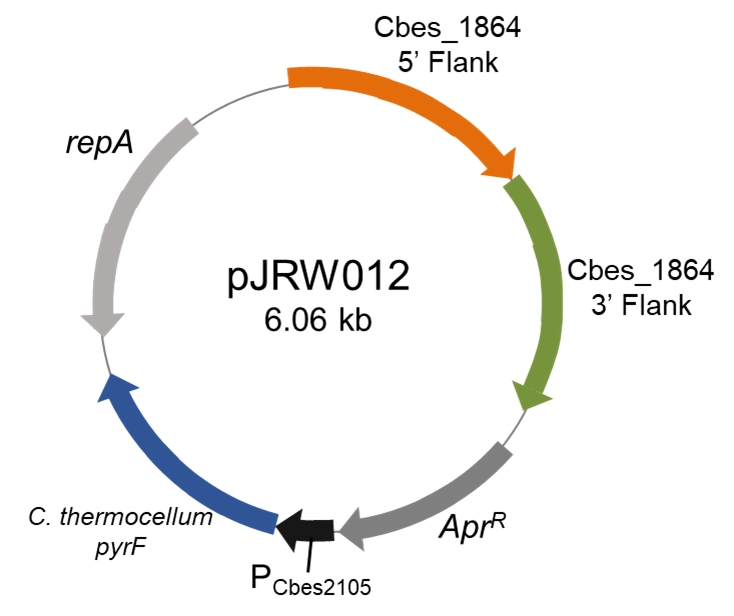


**Figure S1.** Plasmid to target deletion of the Cbes1864 glycosyl transferase gene. A deletion cassette consisting of a fused 5’ and 3’ flanking region was constructed in a non-replicating plasmid, pJRW012. Also labelled are: the apramycin resistance gene cassette (Apr) for selection in E. coli; *repA*, a gene required for pSC101 replication; the pSC101 origin or replication; *par*, the partition locus; and the *pyrF* gene (orotidine 5’-phosphate decarboxylase) from *C. thermocellum* (Clo1313_1266) under the transcriptional control of the regulatory region from ribosomal protein S30EA (Cbes_2105) to allow for the selection of uracil prototrophic transformants of a Δ*pyrF* background strain.


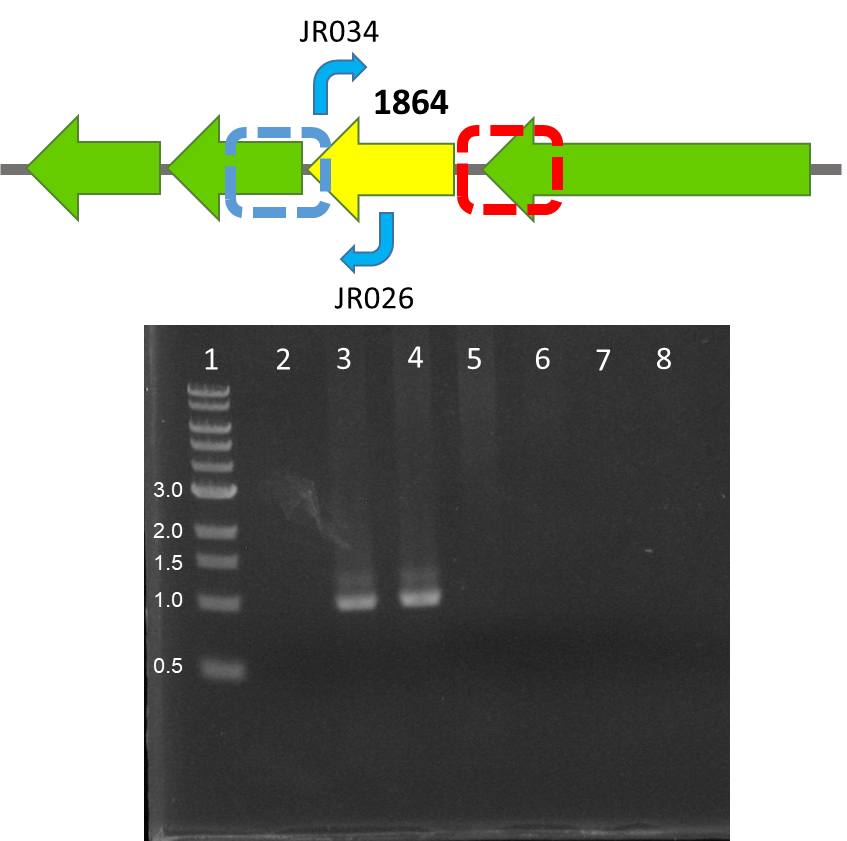


**Figure S2.** Agarose gel showing PCR products amplified with primers JR034 and JR026 on gDNA templates from wild-type *C. bescii* (lane 3), parent strain JWCB018 (lane 4), Δ*cbes1864* strain JWCB143 (lane 5), and no template (lane 2). Lane 1: DNA molecular weight standards, Lanes 6-8: JWCB143 sister isolates. Expected band of 933 bp for the wild-type locus was observed for the WT and parent strains, for Cbes_1864 deletion locus no PCR product was expected or observed. Both primers bind on the Cbes_1864 ORF with JR026 binding within the portion to be deleted and JR034 binding at the C-terminus of the ORF which was not deleted, but was part of the downstream (5’) homologous region used for recombination.


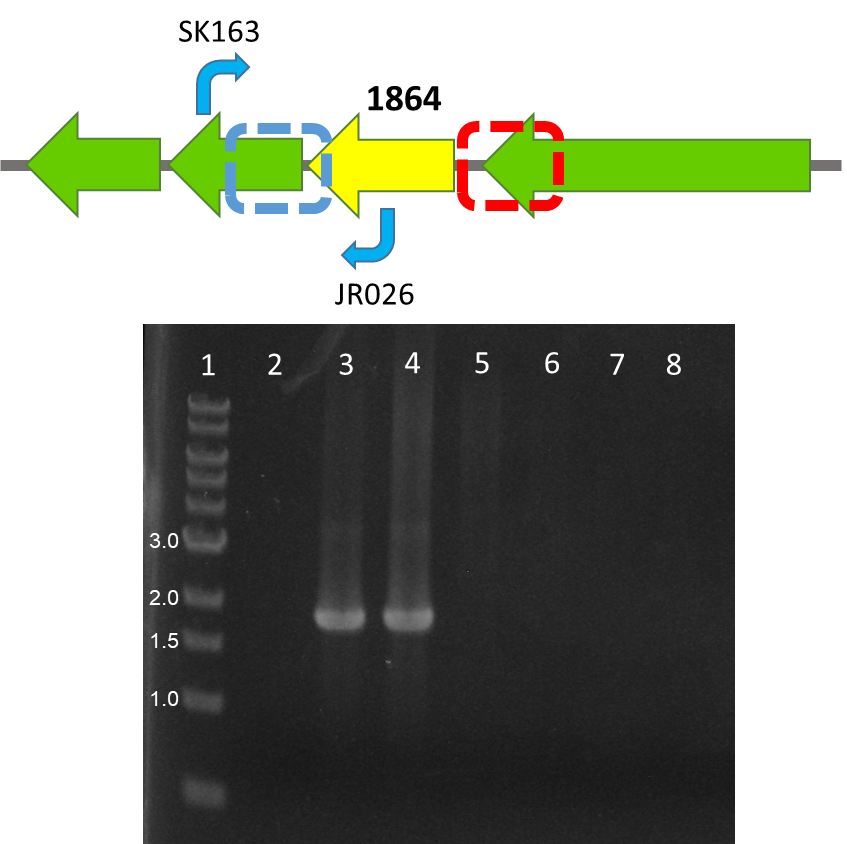


**Figure S3.** Agarose gel showing PCR products amplified with primers SK163 and JR026 on gDNA templates from wild-type *C. bescii* (lane 3), parent strain JWCB018 (lane 4), Δ*cbes1864* strain JWCB143 (lane 5), and no template (lane 2). Lane 1: DNA molecular weight standards, Lanes 6-8: JWCB143 sister isolates. Expected band of 1.8 kb for the wild-type locus was observed for the WT and parent strains and for Cbes_1864 deletion locus no PCR product was expected or observed. JR026 binds within the region of Cbes_1864 targeted for deletion and SK163 binds downstream of the gene and outside of the 5’ downstream flanking region used for recombination.


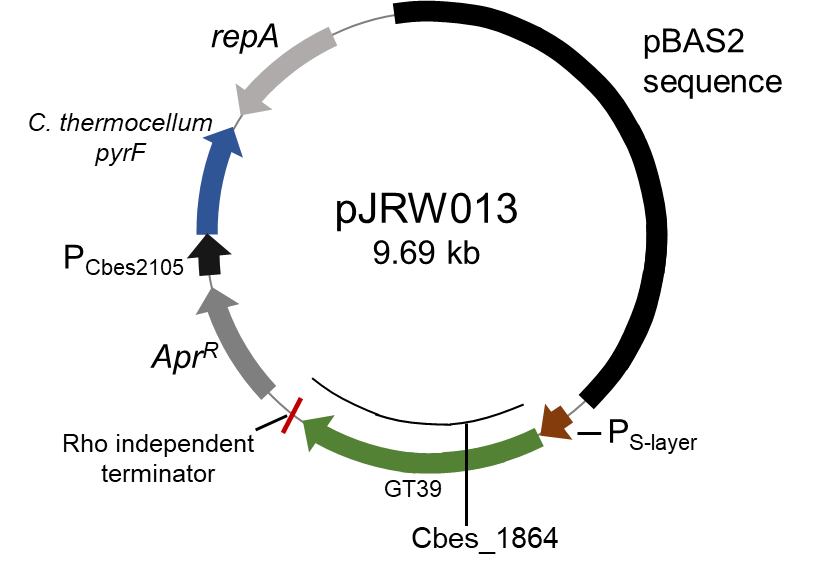


**Figure S4.** Plasmid pJRW013 constructed for expression of the Glycosyl Transferase wild type allele for complementation of the deletion under control of the Cbes_2303 (S-layer protein) promoter. Also labelled are: the apramycin resistance gene cassette (Apr) for selection in E. coli; *repA*, a gene required for pSC101 replication; the pSC101 origin or replication; *par*, the partition locus; the *pyrF* gene (orotidine 5’-phosphate decarboxylase) from *C. thermocellum* (Clo1313_1266) under the transcriptional control of the regulatory region from ribosomal protein S30EA (Cbes_2105) to allow selection of uracil prototrophic transformants of a Δ*pyrF* background strain; and pBAS2 sequence that contains the origin of replication for *C. bescii*.


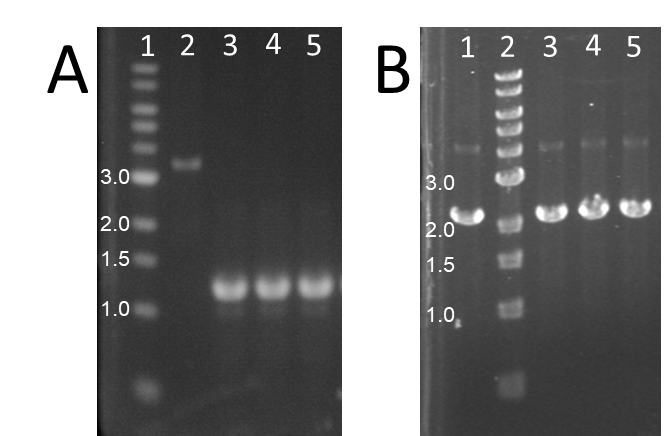


**Figure S5.** Agarose gels showing PCR products confirming strain *C. bescii* JWCB160. **A.** Molecular weight ladder in kbp (lane 1), and PCR products of primers JR026 and DC228 for detection of pJRW013 on the following templates: JWCB143 gDNA (lane 2), JWCB160 gDNA (lane 3), JWCB160 sister isolates gDNA (lanes 4,5). The expected ~1.1 kb band is present only on JWCB160 and sister isolates. **B.** Molecular weight ladder (lane 2), and PCR products of primers SK163 and SK164 on the following templates: JWCB143 gDNA (lane 1), JWCB160 gDNA (lane 3), JWCB160 sister isolates gDNA (lanes 4,5). All lanes contain the ~2.2 kb band expected for the Cbes_1864 deletion locus. The ~3.7 kb band for the wild-type Cbes_1864 locus is not present.
